# Repetitive transcranial magnetic stimulation of the primary motor cortex in stroke survivors-more than motor rehabilitation: A mini-review

**DOI:** 10.3389/fnagi.2022.897837

**Published:** 2022-09-26

**Authors:** Abdulhameed Tomeh, Abdul Hanif Khan Yusof Khan, Wan Aliaa Wan Sulaiman

**Affiliations:** ^1^Department of Neurology, Faculty of Medicine and Health Sciences, Universiti Putra Malaysia, Serdang, Malaysia; ^2^Malaysian Research Institute on Ageing (MyAgeing™), Universiti Putra Malaysia, Serdang, Malaysia

**Keywords:** transcranial magnetic stimulation, primary motor cortex, stroke, motor rehabilitation, non-motor symptoms

## Abstract

Stroke is a leading cause of morbidity and mortality among elderly populations worldwide. During the early phase of stroke, restoring blood circulation is of utmost importance to protect neurons from further injury. Once the initial condition is stabilized, various rehabilitation techniques can be applied to help stroke survivors gradually regain their affected functions. Among these techniques, transcranial magnetic stimulation (TMS) has emerged as a novel method to assess and modulate cortical excitability non-invasively and aid stroke survivors in the rehabilitation process. Different cortical regions have been targeted using TMS based on the underlying pathology and distorted function. Despite the lack of a standard operational procedure, repetitive TMS (rTMS) of the primary motor cortex (M1) is considered a promising intervention for post-stroke motor rehabilitation. However, apart from the motor response, mounting evidence suggests that M1 stimulation can be employed to treat other symptoms such as dysphagia, speech impairments, central post-stroke pain, depression, and cognitive dysfunction. In this mini-review, we summarize the therapeutic uses of rTMS stimulation over M1 in stroke survivors and discuss the potential mechanistic rationale behind it.

## Introduction

Stroke is defined as an episode of acute neurological impairment caused by an ischemic infarction or hemorrhage resulting in focal injury to the brain (Sacco et al., 2013). With an estimated ≈ 13 million cases reported annually, stroke is considered one of the major causes of neurological disability worldwide (Saini et al., 2021). Despite advances in stroke management, many stroke survivors suffer from long-term residual effects (Chohan et al., 2019).

Transcranial magnetic stimulation (TMS) has emerged as a painless, non-invasive technique that can stimulate the human brain and aid stroke survivors in rehabilitation after stroke (Hallett, 2007). Therapeutically, TMS is delivered in a repetitive manner with various stimulation parameters in terms of intensity, frequency, and number of sessions. The mechanism of action of repetitive TMS (rTMS) is believed to rely on principles of long-term potentiation/depression (LTP/LTD)- synaptic plasticity that has been extensively studied at the cellular level (Huang et al., 2017). Excitatory protocols consist of high-frequency (5–20 Hz) rTMS and ultra-high frequency (50 Hz) rTMS applied in the form of a patterned protocol called intermittent theta-burst stimulation (iTBS), while inhibitory protocols consist of low-frequency (≤ 1 Hz) rTMS and continuous TBS (cTBS) (Hallett, 2007). As stroke survivors frequently encounter motor impairments with slow recovery, rTMS stimulation of the primary motor cortex (M1) has been widely investigated to promote the motor rehabilitation process with encouraging results.

However, M1 stimulation has shown therapeutic efficacy beyond the conventional motor symptoms to involve dysphagia, speech impairments, central post-stroke pain, depression, and cognitive dysfunction. This mini-review aims to summarize the latest knowledge about M1 stimulation in post-stroke rehabilitation and discuss the mechanistic rationale behind the management of motor and non-motor symptoms.

## Motor rehabilitation

The motor impairment after stroke was shown to result from the damaged corticospinal output from the affected M1 along with transcallosal inhibitory drive from the unaffected M1 (Ward and Cohen, 2004). The latter effect is based on the concept of dysbalanced interhemispheric interaction where the unaffected (contralesional) hemisphere becomes “overactive” and slows the recovery process of the affected (ipsilesional) hemisphere (Ward and Cohen, 2004). Therefore, excitatory (high-frequency) rTMS protocols are usually applied over the ipsilesional hemisphere, whereas inhibitory (low-frequency) rTMS protocols are applied over the contralesional hemisphere to restore the interhemispheric balance. A recent meta-analysis revealed that the high-frequency rTMS over the ipsilesional M1 enhanced its excitability without affecting that of the contralesional M1. On the other hand, the low-frequency rTMS protocol not only decreased the contralesional M1 excitability but also enhanced that of the ipsilesional M1, further supporting the bimodal balance recovery model (Bai et al., 2022).

### Rehabilitation of upper limbs

Rehabilitation of upper limbs after stroke has been more extensively studied than the lower limbs, presumably due to the easiness of TMS application over the M1 hand homunculus (Rossini et al., 2015). High-quality evidence from recent systematic reviews and meta-analysis has shown that rTMS over M1 significantly improved the upper limb impairments, including fine motor movements, grip strength, and activities of daily living (O’Brien et al., 2018; He et al., 2020), without affecting spasticity levels significantly compared to sham stimulation (McIntyre et al., 2018; Xu P. et al., 2021).

The improvement in motor recovery was sustained for at least 1 month after 5 daily sessions of the rTMS (Zhang et al., 2017). Whereas delivering more than 5 sessions in one meta-analysis (Zhang et al., 2017), or 7 in another (Xiang et al., 2019), did not yield additional improvement in the upper limb recovery. On the other hand, patients with pure subcortical strokes were found to benefit better from the rTMS therapy compared to those with cortical strokes (Zhang et al., 2017; Xiang et al., 2019).

In relation to stimulation frequency, both low- and high-frequency rTMS protocols were effective in the motor recovery after stroke (Zhang et al., 2017; Xiang et al., 2019). While in TBS protocols, iTBS over the ipsilesional M1 was thought to yield better efficacy on the upper limb recovery than cTBS over the contralesional M1 (Zhang et al., 2017), a finding that an ongoing meta-analysis will further investigate with the inclusion of more accumulating TBS studies (Liu et al., 2019). On the other hand, a recent large network meta-analysis has revealed by probability ranking that among all the aforementioned protocols, the high-frequency (≥ 10 Hz) rTMS may be the most effective protocol for improving the upper limb motor function in stroke patients (Xia et al., 2022).

Considering the optimal time window, applying rTMS as early as the first month after stroke has proved to be more beneficial in promoting the upper limb function in comparison to subacute (1–6 months) and chronic (> 6 months) phases of stroke (van Lieshout et al., 2019). This is consistent with neurophysiological and histological findings in patients and animal models showing that there is an early critical time window during which the brain in more likely to be responsive to neurorehabilitation treatments (Krakauer et al., 2012). In addition, it has been shown that most recovery processes take place within the first 3 months following a stroke, after which improvement is thought to reach a plateau phase (Biernaskie et al., 2004; Krakauer et al., 2012).

Noteworthy, the rTMS stimulation in motor rehabilitation is thought to optimize the effects of other interventions rather than provide the brain with all the changes needed for motor skill acquisition. Therefore, rTMS was usually employed in combination with conventional physiotherapy and occupational therapy (Ahmed et al., 2022). In addition, novel combinations have been investigated simultaneously with the rTMS application, including robotic training (Di Lazzaro et al., 2016; Miller et al., 2019) and virtual reality (Zheng et al., 2015) with promising results.

### Rehabilitation of lower limbs

Evidence from recent systematic reviews and meta-analyses has shown that rTMS stimulation of the lower limbs representation at M1 significantly enhanced the motor recovery in stroke patients, including walking speed, spasticity, functional balance, and postural control (Tung et al., 2019; Kang et al., 2020; Liu Y. et al., 2021; Krogh et al., 2022). The number of daily sessions ranged between 5–20 sessions, with the higher number of sessions resulting in a cumulative improvement in the treatment effects (Kang et al., 2020).

Concerning stimulation frequency, low-frequency rTMS over the contralesional M1 seemed to improve the lower limb function better than high-frequency rTMS over the ipsilesional M1, according to a recent network meta-analysis (Xie et al., 2021). On the other hand, while iTBS was more effective than sham stimulation in promoting lower limb recovery, data from cTBS studies are lacking (Xie et al., 2021).

In regard to the optimal timing, rTMS application during the subacute (1–6 months post-stroke) and chronic phases (> 6 months post-stroke) is supported by the current evidence to benefit balance and gait recovery, with limited data on the acute phase (< 1 month after stroke onset) (Parikh et al., 2021).

## Dysphagia

Dysphagia is a common complication in stroke patients and could result in aspiration pneumonia, malnutrition, and dehydration (Takizawa et al., 2016). Different neuromodulation techniques have been investigated to promote swallowing recovery after stroke, including transcranial direct current stimulation, surface neuromuscular electrical stimulation, and pharyngeal electrical stimulation. However, rTMS stimulation was superior to these techniques in the swallowing recovery based on the results of two recent network meta-analyses (Chiang et al., 2019; Li et al., 2021). The mechanism of action of M1-rTMS on dysphagia rehabilitation depends on enhancing neuroplasticity of the disrupted corticobulbar neural pathways that project to swallowing muscles (Gow et al., 2004).

Stimulation targets of the M1 have varied between the tongue, mylohyoid, pharyngeal, and esophageal representations at the M1 (Yang et al., 2021). In addition, no significant difference in the swallowing recovery was noted between low- and high-frequency rTMS protocols (Yang et al., 2021). Nonetheless, bihemispheric M1-rTMS has shown better effects on the swallowing function compared to unilateral stimulation (Cheng et al., 2020). In addition, it’s recommended to combine rTMS with traditional swallowing rehabilitation training to achieve better outcomes (Dziewas et al., 2021).

Concerning the optimal time window, the effect of therapeutic rTMS was most beneficial when applied within the first 2 weeks of stroke, and the effects were most substantial during the first 2 months following application (Cheng et al., 2020).

## Speech rehabilitation

Based on the cortical region affected, stroke can result in impaired speech production in the form of aphasia or dysarthria (Schindel et al., 2022). As a therapeutic approach, rTMS was mainly applied at the inferior frontal gyrus corresponding to Broca’s area in aphasia rehabilitation (Arheix-Parras et al., 2021). Recently, however, a concurrent use of iTBS and functional magnetic resonance imaging (fMRI) has proposed a therapeutic potential for the iTBS protocol targeting the affected M1 at the hand representation in patients with post-stroke aphasia (Xu S. et al., 2021). These results were in accordance with previous reports showing functional connectivity between the cortical language network and the M1 hand representation (Meister et al., 2003, 2006; Meinzer et al., 2016). While in patients with post-stroke dysarthria, applying low-frequency rTMS to the unaffected mouth representation of M1 in combination with speech therapy improved the articulation functionality significantly in comparison to speech therapy alone (Kwon et al., 2015).

## Central post-stroke pain

Central post-stroke pain (CPSP) is defined as a pain occurring after a cerebrovascular lesion of the brain or brainstem (Scholz et al., 2019). This pain is felt in the body region corresponding to the central nervous structure affected by stroke (Scholz et al., 2019). It is estimated that more than 50% of patients with a stroke affecting the somatosensory tract will develop CPSP (Liampas et al., 2020). The pathophysiology of CPSP is related to a defective gamma-aminobutyric acid (GABA)-ergic inhibition inside the brain, leading to maladaptive plasticity in multiple cortical regions, including M1 (Tang et al., 2019). Therefore, many studies have targeted the M1 cortex with rTMS to restore the intracortical inhibition in patients with CPSP. As a result, a direct relationship was noted between the analgesic efficacy and the modulation of M1 cortical excitability (Lefaucheur et al., 2006; Hosomi et al., 2013). In addition, the analgesic efficacy of M1 stimulation is believed to stem from the interconnection between M1 and the endogenous opioid system. This relation was evidenced by positron emission tomography scans (Maarrawi et al., 2007; Lamusuo et al., 2017) and pharmacological blocking of the μ-opioid receptors, which minimized the analgesic efficacy of M1 stimulation (de Andrade et al., 2011). On the neural network level, M1 stimulation was found to modulate the excitability of other cortical and subcortical areas related to sensory, cognitive, and emotional components of pain, such as the thalamus, insular cortex, and anterior cingulate gyrus (García-Larrea et al., 1999; Hasan et al., 2014). Recent systematic reviews have shown that 5–10 sessions of high-frequency rTMS over M1 of the affected hemisphere resulted in a significant reduction in the CPSP intensity which lasted for at least 3 weeks post-treatment (Ramger et al., 2019; Liampas et al., 2020).

## Depression

Depression is a common complaint after stroke, affecting approximately 30% of stroke survivors (Towfighi et al., 2017). Patients with post-stroke depression are at higher risk of recurrent strokes and mortality (Towfighi et al., 2017). Previous rTMS studies have mainly targeted the dorsolateral prefrontal cortex as a therapeutic approach in patients with post-stroke depression (Shen et al., 2017). However, preliminary evidence has shown that low-frequency stimulation of the contralesional M1 cortex might have an antidepressant effect in depressed stroke patients (Carey et al., 2008; Niimi et al., 2020). The mechanistic rationale for the antidepressant efficacy of M1 stimulation might stem from its effect on the kynurenine levels (Kepplinger et al., 2014; Niimi et al., 2020), a tryptophan metabolite and one implicated pathway in depression (Ogyu et al., 2018). In addition, a recent meta-analysis combined with functional magnetic resonance imaging studies has identified M1 as a region of interest (ROI) for rTMS in depressive disorders (Zhang et al., 2020). Notably, some individual studies have reported an antidepressant efficacy following high-frequency rTMS at unilateral or bilateral M1 in Parkinson’s disease and chronic pain conditions (Khedr et al., 2015; Makkos et al., 2016; Li et al., 2020; Bursali et al., 2021). On the other hand, the antidepressant efficacy was not found in other studies with similar conditions and stimulation parameters (Brys et al., 2016; Lindholm et al., 2016; Hosomi et al., 2020). However, these findings need to be further explored in larger randomized controlled studies. In addition, it is worth mentioning that the emotional improvement following rTMS at M1 might be an indirect effect of the concomitant improvement in other symptoms, such as motor symptoms and pain.

## Cognitive impairment

It is estimated that up to 83% of stroke survivors suffer from cognitive impairment in at least one cognitive domain (Jokinen et al., 2015). Similar to depression, the dorsolateral prefrontal cortex has been a conventional target for rTMS in patients with post-stroke cognitive impairments (Liu M. et al., 2021). However, mounting evidence suggests that the M1 is directly involved in higher cognitive processes including, but not limited to, attention, memory, motor imagery, and language comprehension (Tomasino and Gremese, 2016; Vukovic et al., 2017; Bhattacharjee et al., 2021; Vitale et al., 2021). In this context, two recent studies have shown that low-frequency rTMS stimulation over the contralesional M1 improved the measures of global cognition and visuospatial recall memory in stroke patients (D’Agata et al., 2016; Askin et al., 2017). Furthermore, this improvement was associated with reduced latency in the N200 and P300 markers of event-related potentials, indicating an increasing speed in the perceptual and cognitive processes (D’Agata et al., 2016). Noteworthy, the improvement in measures of global cognition along with the reduced latency in P300 following high-frequency bilateral M1-rTMS has also been reported in patients with Parkinson’s disease-related dementia (Khedr et al., 2020). This, in turn, might suggest a consistent procognitive effect of the M1-rTMS regardless of the underlying pathology.

## Conclusion and perspective

With the slow and often incomplete recovery from stroke sequelae, rTMS is becoming an increasingly used technique in post-stroke rehabilitation. Stimulation of the M1 cortex has long been employed to treat residual motor symptoms of stroke incidents. However, accumulating evidence suggests that M1 stimulation can ameliorate a variety of non-motor symptoms that stroke patients frequently experience. The mechanistic rationale behind the management of these symptoms varies depending on the neural networks involved in their pathophysiology. Nonetheless, whether applied in the acute or chronic phase, alone or in combination with other interventions, rTMS stimulation over M1 can yield a therapeutic efficacy that extends beyond the movement execution to involve swallowing, speech, pain, mood, and cognition. Therefore, well-conducted randomized controlled trials, in particular studies combining TMS with EEG and neuroimaging techniques, will help expand our knowledge of the cortical and subcortical connections of M1 in health and disease and ultimately tailor the therapeutic use of rTMS based on the constellation of symptoms in each patient. In addition, as we approach the personalized medicine era, accumulating evidence highlights the influence of genetic polymorphisms on the rate of stroke recovery (Math et al., 2019). This might explain, in part, the variability in responses to the TMS in post-stroke rehabilitation. Therefore, future studies should further explore the potential genetic polymorphisms that interact with TMS responses in stroke, bearing in mind that the most investigated one, the brain-derived neurotrophic factor, has not yet proven as a decisive factor in the M1-rTMS literature (Sasaki et al., 2021).

## Author contributions

AT devised the idea for this review and prepared the initial manuscript draft. AT, AHKYK, and WAWS wrote, edited, and revised the final manuscript. All authors approved the final version of the manuscript before submission.
